# Understanding Cognitive Deficits in People with Coronary Heart Disease (CHD)

**DOI:** 10.3390/jpm13020307

**Published:** 2023-02-10

**Authors:** Weixi Kang, Antonio Malvaso

**Affiliations:** 1 UK DRI Care Research and Technology Centre, Department of Brain Sciences, Imperial College, London W12 0BZ, UK; 2 IRCCS “C. Mondino” Foundation, National Neurological Institute, Department of Brain and Behavioral Sciences, University of Pavia, 27100 Pavia, Italy

**Keywords:** coronary heart disease, cognitive deficits, episodic memory, semantic verbal fluency, fluid reasoning, numerical ability

## Abstract

Coronary heart disease (CHD) is one of the main cardiovascular diseases that can cause disability and death across the globe. Although previous research explored the links between CHD and cognitive deficits, only a subset of cognitive abilities was analyzed and a small clinical sample size was used. Thus, the aim of the current study is to assess how CHD can affect the cognitive domains of episodic memory, semantic verbal fluency, fluid reasoning, and numerical ability in a large cohort of participants from the United Kingdom. Results revealed that episodic memory, semantic verbal fluency, fluid reasoning, and numerical ability are negatively affected by CHD. Prevention and intervention should be developed to preserve cognitive abilities in people with CHD, but more studies should explore specific ways of doing so.

## 1. Introduction

As one of the main cardiovascular diseases, coronary heart disease (CHD) is the world’s leading cause of disability and death [1]. CHD is characterized by a lot of clinical symptoms, manifested as angina, myocardial infarction (MI), and/or sudden cardiac death [2,3]. 

Cognitive deficits are very common among older people, which are characterized by a lower performance on a wide range of cognitive domains, such as attention, memory, and executive function, even after accounting for age and educational level [4]. Dementia is a state of disease in which cognitive deficits are severe enough to affect normal functioning [5]. According to studies, the global prevalence of dementia ranges between 5% and 7%, with the highest rate in developing countries [5]. Cognitive impairments impose significant individual, societal, and financial burdens on people, especially as the world’s population ages [6]. 

Regarding the associations between CHD and cognitive impairments, some previous studies found that CHD is a major risk factor of cognitive decline [7,8], whereas others found no link between cognitive impairments and CHD [9,10]. A more recent meta-analysis found a link between CHD and the risk of cognitive impairment [11]. Subgroup findings also revealed that people with CHD are at a higher risk of developing vascular dimensions, but not Alzheimer’s disease [11,12]. 

There are several reasons that can explain why CHD could negatively affect cognition, which may include (1) common risk factors of both CHD and cognitive impairments, such as low physical activities, (2) CABG surgery, which may affect cognitive abilities in people with CHD, and (3) inflammation associated with CHD (see [11]).

Thus, the relationship between CHD and cognitive impairments remains unclear given that the variability in these studies primarily involved small clinical samples. Therefore, there is a need for cohort-based studies, which are characterized by high generalizability. In addition, cognition is not a unitary concept but has multiple components. Previous studies focused on one or more cognitive domains in one study but ignored others. Thus, the aim of the current study is to assess how CHD can affect the cognitive domains of episodic memory, semantic verbal fluency, fluid reasoning, and numerical ability in a large cohort of participants from the United Kingdom.

## 2. Methods

### 2.1. Data

We extracted and analyzed data from Understanding Society: collected annual data from a representative sample of UK households since 1991, the UK Household Longitudinal Study (UKHLS) [13]. Participants were asked whether they have been clinically diagnosed with CHD in Wave 1 (collected between 2009 and 2010). After that, participants were asked if they had been clinically diagnosed with CHD in each wave until Wave 3. Participants completed cognitive measures at Wave 3 (collected between 2011 and 2012). We removed individuals with any missing fields of interest. Age- and sex-matched healthy controls were chosen from the people who indicated that they were not clinically diagnosed with CHD. Thus, there were 260 participants with clinically diagnosed CHD, with a mean age of 63.61 ± 16.19 (61.15% males), and 4782 participants without CHD with a mean age of 63.40 ± 9.90 (61.36% males).

### 2.2. Measures

#### 2.2.1. CHD

In population-based studies, the validity of self-reported cardiovascular disease has been approved (e.g., [14]). The question “Has a doctor or other health professional ever told you that you have any of these conditions?” was used to assess CHD in retrospect at Wave 1. Following-up waves asked participants if they had recently been diagnosed with CHD.

#### 2.2.2. Cognitive Abilities

Episodic memory was assessed with immediate and delayed word recall tasks. An animal fluency task was used to assess semantic verbal fluency [15,16,17]. The number series task is intended to assess fluid reasoning, or the ability to solve novel problems using abstract thought. Logic puzzles are commonly used to assess fluid reasoning [15]. Numerical ability tests involve solving problems that might be encountered in daily life, which was measured by a series of questions such as “In a sale, a shop is selling all items at half price. Before the sale, a sofa costs £300. How much will it cost in the sale?” Details of the procedures of these tasks can be found at: https://www.understandingsociety.ac.uk/sites/default/files/downloads/documentation/mainstage/user-guides/6614_Cognitive_Ability_measures_v1.1.pdf (accessed on 10 January 2022) and were copied below. All scores were standardized before further analysis (mean = 0, S.D. = 1).

The immediate and delayed recall tasks: “For this task, the computer reads a list of 10 words to standardise the presentation and speed of the word list. The interviewer checks if the respondent can hear the computer playing a short test message. If the voice cannot be heard, the interviewer checks again, following adjustment of the volume. If the respondent still cannot hear the computer’s voice, the interviewer reads the words at a slow steady rate of about one word every two seconds. The list of words is not repeated. No aids are allowed for the test. Interviewer: The computer will now read a set of 10 words. I would like you to remember as many as you can. We have purposely made the list long so it will be difficult for anyone to remember all the words. Most people remember just a few. Please listen carefully to the set of words, as they cannot be repeated. When it has finished, I will ask you to recall aloud as many of the words as you can, in any order. Is this clear? Now please tell me the words you can remember. Respondents give the words in any order. The interviewer codes each correct response. For the delayed word recall test, after the Number Series test (below), respondents were again asked to remember the words from the list. The interviewer codes each correct response. We used the word lists developed for the HRS, as does ELSA. The different lists were given to members of the same household based on random assignment. The lists can also be varied in subsequent waves to reduce learning.”

Animal naming task: “Interviewer: Now, I would like you to name as many animals as you can. You have one-minute, so name them as quickly as possible. We will begin when you say the first animal. If you are unsure of anything, please ask me now, as I am unable to answer questions once the minute starts. The interviewer instructions are to write down the actual words in the order in which they are produced. They are recorded in the Cognitive Ability Booklet. With respect to scoring, extinct, imaginary or magical (e.g., dodo, unicorn, dragon) animals were scored as correct, but given names (e.g., Felix, Buster) were not. The assessment was timed by CAPI. The interviewer began the 60 s countdown on the computer as soon as the respondent said the first correct word.”

Number series task: “For this test, respondents use a pencil and paper to write down the number sequences as read by the interviewer. The number series consists of several numbers with a blank number in the series. The respondent will be asked which number goes in the blank. The interviewer begins with a simple example so the respondent can see how the test works. For the example, the interviewer can tell the respondent if they give an incorrect response and inform them of the correct answer. If the respondent does not understand the instructions, or answered ‘Don’t know’ in the example, a further example is worked through. If they answer incorrectly a second time, CAPI instructs the interviewer to inform them of the correct response and explain how the sequence works. If the respondent still does not understand, or seems confused, the interviewer codes this and moves on to the next task. However, if the respondent understands the task, the interviewer moved on to the number series.”

Numerical ability test: “Interviewer: Next, I would like to ask you some questions to understand how people use numbers in everyday life. If CATI, the interviewer added: You might want to have a pencil and paper handy to help you answer the following items. The measure of numeric ability asks respondents up to five questions that are graded in complexity. Based on performance on the first three items, respondents can get additional more difficult items and a higher score, or an additional more simple item. ‘Don’t know’ was not a permitted response. There was a showcard with the text of the question. This can be seen in the fieldwork documents: https://www.understandingsociety.ac.uk/documentation/mainstage/fieldworkdocuments (accessed on 1 January 2020).”

#### 2.2.3. Demographic Controls

Demographic controls included age, sex, monthly income, highest educational qualification, legal marital status, and residence. The way these variables were coded in the model can be found in Table 1.

### 2.3. Analysis

A train-and-test approach was used to analyze this dataset. First, five generalized linear models were constructed based on data from people without CHD, by taking demographic controls in the model including age, sex, monthly income, highest educational qualification, legal marital status, and residence into the model as independent variables to predict outcome cognitive variables, including the delayed word recall, the immediate word recall, numeracy, and the number series scores. Then demographics from people with CHD were input into these models to estimate the expected scores of people with CHD, as if they have not been clinically diagnosed with CHD. Finally, one-sample t-tests were used to determine the differences between the expected and actual cognitive scores of people with CHD. This approach has more advantages than paired-sample t-tests, because it does not require equal same size and can control potential confounders, such as demographics.

## 3. Results

**Figure 1:**
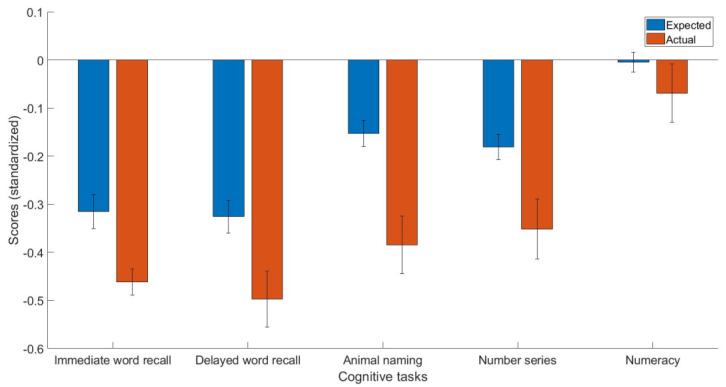
The expected and actual cognitive task scores in people with CHD.

Descriptive statistics can be found in Table 1. The estimates (*b*) of the linear models trained on healthy controls can be found in Table 2. The main findings were that people with CHD are characterized by a lower immediate word recall (t(259) = −2.81, *p* < 0.01, Cohen’s d = −0.15, 95% C.I. = [−0.25, −0.04]), delayed word recall (t(259) = −3.31, *p* < 0.01, Cohen’s d = −0.17, 95% C.I. = [−0.27, −0.07]), semantic verbal fluency (t(259) = −3.82, *p* < 0.001, Cohen’s d = −0.23, 95% C.I. = [−0.35, −0.11]), and fluid reasoning (t(259) = −2.96, *p* < 0.01, Cohen’s d = −0.17, 95% C.I. = [−0.28, −0.06]), compared to what they would expect given their demographics (Figure 1).

## 4. Discussion

Taken together, the current study’s goal is to assess how CHD can affect the cognitive domains of episodic memory, semantic verbal fluency, fluid reasoning, and numerical ability in a large cohort of participants from the United Kingdom. Results revealed that episodic memory, semantic verbal fluency, fluid reasoning, and numerical ability are negatively affected by CHD, which appears to be in line with several previous studies [7,8,11,12], but inconsistent with others that did not find such associations [9,10,12].

However, the potential underlying mechanisms that link CHD and cognitive impairments are still unclear. Previous studies have found several common risk factors for CHD and cognitive impairments among older people, including low physical activity, CHD mellitus, and hypertension [18,19,20,21,22,23]. Thus, the widely acknowledged cardiovascular risk factors may contribute to the development of cognitive impairments. In addition, studies have found that multiple comorbid cardiovascular risk factors contribute to dementia [23,24,25]. Specifically, Bleckwenn et al. (2017) found that CHD influences cognitive decline in people who have recently been diagnosed with dementia [26]. Atrial fibrillation represents one of the most common cardiac arrhythmias, with its prevalence estimated to be ranging from 0.2 to 5% in CHD patients. After coronary artery bypass graft (CABG) surgery, arrhythmia is another common symptom, occurring in approximately 20 to 40% of patients [27]. Atrial fibrillation has been identified as a significant risk factor of cognitive impairment [28,29]. Moreover, CABG surgery is also related to cognitive impairments, with around 50% of patients with cognitive impairment after 1 to 5 years post-surgery [30]. Atherosclerosis progression is closely connected to CHD and negatively affects cerebral vessels, resulting in cerebrovascular dysfunctions and cerebral blood flow impairments [31,32], which may, then, contribute to vascular dementia. Inflammation associated with CHD may also play a role in the cognitive impairment of people with CHD [33,34].

There are some limitations to our study, as well. First, cross-sectional studies cannot establish a causal relationship. A longitudinal approach should be used in future studies to determine the temporal order of CHD diagnosis and cognitive impairments. Second, cardiovascular risk factors, treatment, and medication were not controlled in our study, given that they might have had an influence on the results. Future studies should ascertain information collection about treatment and medication. Third, we did not control for mental health comorbidities, which may have an effect on cognition. Future studies should try to do so. Finally, our study only included people with CHD from the United Kingdom, making it difficult to make assumptions about different countries. Future studies on how CHD can affect cognitive abilities in other countries should be conducted.

In conclusion, we looked at how CHD can affect the cognitive domains of episodic memory, semantic verbal fluency, fluid reasoning, and numerical ability in a large cohort of participants from the United Kingdom. Results revealed that episodic memory, semantic verbal fluency, fluid reasoning, and numerical ability are negatively affected by CHD. However, we were unable to rule out the effect of confounders, such as cardiovascular factors, treatment, and medication. Prevention and intervention should be developed to preserve cognitive abilities in people with CHD, but more studies should explore specific ways of doing so.

## Figures and Tables

**Table 1:** Descriptive statistics of demographic characteristics and cognitive performance for people with and without CHD.

	People with CHD		People without CHD	
	Mean	S.D.	Mean	S.D.
Age	63.61	16.19	63.40	9.90
Immediate task recall	1344.03	1343.91	1598.97	1657.20
Delayed task recall (standardized)	−0.46	0.93	−0.28	0.95
Semantic verbal fluency (standardized)	−0.50	0.96	−0.30	0.94
Fluid reasoning (standardized)	−0.38	1.01	−0.12	0.98
Numerical ability (standardized)	−0.35	0.98	−0.13	1.00
	N	%	N	%
**Sex**				
Male	159	61.15	2934	61.36
Female	101	38.85	1848	38.64
**Highest educational qualification**				
Below college	204	78.46	3523	73.67
College	56	21.54	1259	26.33
**Legal marital status**				
Single	99	38.08	1656	34.63
Married	161	61.92	3126	65.37
**Residence**				
Urban	194	74.62	3426	71.64
Rural	66	25.38	1356	28.36

**Table 2:** The estimates (*b*) of linear models trained based on demographic predictors.

	Intermediate Word Recall	Delayed Word Recall	Animal Naming	Number Series	Numeracy
Age	−0.03 **	−0.03 ***	−0.02 ***	−0.02 ***	−0.01 ***
Sex	0.26 ***	0.25 ***	0.07 **	−0.19 ***	−0.29 ***
Monthly income	0.00 ***	0.00 ***	0.00 ***	0.00 ***	0.00 ***
Highest educational qualification	0.36 ***	0.29 ***	0.27 ***	0.50 ***	0.41 ***
Legal marital status	0.02	0.01	0.10 ***	0.16 ***	0.13 ***
Residence	0.08 **	0.05	0.11 ***	0.11 ***	0.07 **
R^2^	0.18	0.16	0.10	0.15	0.15

All numbers are rounded up to two decimal places. ** *p* < 0.01 *** *p* < 0.001.

## Data Availability

Publicly available datasets were analyzed in this study. This data can be found here: https://www.understandingsociety.ac.uk (accessed on 7 February 2022).
